# Chemical Speciation and Characterization of Trace Metals in Dry Camellia sinensis and Herbal Tea Marketed in Nigeria

**DOI:** 10.5696/2156-9614-8.19.180912

**Published:** 2018-09-10

**Authors:** Omowunmi H. Fred-Ahmadu, Adebusayo E. Adedapo, Mary O. Oloyede, Nsikak U. Benson

**Affiliations:** Analytical and Environmental Chemistry Unit, Department of Chemistry, Covenant University, Ota, Ogun State, Nigeria

**Keywords:** tea, *Camellia sinensis*, trace metals, herbal tea, chemical fractionation

## Abstract

**Background.:**

Trace metals from anthropogenic activities have been found to occur in tea brands and pose potential human health risks to consumers.

**Objectives.:**

The present study assessed the concentrations of trace metals in green, black and herbal tea brands using a modified Community Bureau of Reference sequential extraction method.

**Methods.:**

Fifteen (15) *Camellia sinensis* and eight (8) herbal tea samples commonly consumed in Nigeria were collected and analyzed for trace metals. The concentrations of cadmium (Cd), chromium (Cr), copper (Cu), manganese (Mn), nickel (Ni), lead (Pb), vanadium (V), and zinc (Zn) in extract fractions were analyzed using microwave plasma atomic emission spectroscopy (MP-AES).

**Results.:**

Trace metals were detected in all of the samples investigated. The concentrations of trace metals in 4 stages (soluble/exchangeable/carbonates bound fraction, reducible fraction, oxidizable fraction, residual fraction) of sequential and pseudo-total metal extraction procedures are presented. The concentrations of Cd, Cr, Cu, Mn, Ni, Pb, V, and Zn in the exchangeable/carbonate bound fraction for green tea ranged between 0.27–1.47, ND-0.33, ND-0.44, 7.05–33.04, 0.23–0.69, ND-0.51, ND-0.16 and 0.18–1.99 mg/kg, ND-0.73, 0.15–0.36, 0.36–0.59, 1.38–30.07, 0.15–0.54, 0.05–0.76, 0.15–0.34 and 0.27–0.77 mg/kg and 0.54–0.64, 0.25–0.41, 0.35–0.47, 18.72–23.98, 0.30–0.55, 0.15–0.21, 0.15–0.23 and 0.30–0.48 mg/kg for hebal tea, respectively.

**Conclusion.:**

The metal content in the investigated tea indicated low to enhanced concentrations. Locally produced black teas recorded relatively low trace metal contents compared to the green and herbal tea samples. The most bioavailable trace metal was Mn, while Zn was most preferably bound to the residual fraction. Cadmium, Cr, Cu, Ni, Pb, and V were distributed at varied concentrations among other extractable phases. Daily consumption of the investigated tea products may expose consumers to potentially toxic metals as well as essential elements.

**Competing Interests.:**

The authors declare no competing financial interests.

## Introduction

Trace metals are chemical elements which naturally occur at low concentrations in the environment and are required at very low levels in living systems, but are toxic at enhanced levels.^1–3^ This includes elements such as cadmium (Cd), chromium (Cr), cobalt (Co), copper (Cu), iron (Fe), lead (Pb), molybdenum (Mo), manganese (Mn), nickel (Ni), and zinc (Zn). Elemental contamination has been of growing concern in environmental matrices such as soil, sediment, water, medicinal plants, fruits and vegetables, and particulate matter in the atmosphere.^1–15^ Foodstuff, beverages and consumer products like cigarettes have been shown to have enhanced levels of toxic trace metals.^16–18^ Essential trace elements including Cu, Fe, Mn and Zn are required for the proper functioning of enzymes in the body; however, they become toxic when their concentrations are enhanced and begin to accumulate in body tissues. Nonessential trace metals such as Cd and Pb are toxic to the human body even at low concentrations. In other related reports, low and high molecular weight polycyclic aromatic hydrocarbons (PAHs) were detected in commercially sold green tea and herbal products.^19^,^20^

Tea (black and green) is widely produced from the plant of the Theaceae family known as Camellia sinensis. It is a commonly consumed beverage across the world, and is widely shown to have immense benefits such as the supply of dietary requirements of trace elements to the human body, stimulant properties, cardiovascular disease risk reduction, weight management, antioxidant capacity, and control of type II diabetes.^21^ It is considered to be an important source of vital elements like calcium (Ca) and potassium (K), essential trace elements, and toxic trace elements.^22^ Habitual tea consumption can contribute to daily human dietary requirements, but it can also be a source of increased levels of toxic trace metals like arsenic (As), aluminum (Al), Cd, and Pb. Over the years, several methods have been developed for processing different types of tea and are primarily differentiated by the extent of fermentation or oxidation of the tea leaves. Tea types include the non-oxidized and non-fermented process to produce the green tea, and the fully oxidized and fermented method, which results in the production of black tea. Herbal teas are widely produced from well-dried, ground (in some products), and processed roots, stem bark, seeds, or flowers of herbaceous plants, and may not necessarily contain Camellia sinensis leaves. Usually, the oxidative processing involves the transformation of flavan-3-ols to theaflavins and thearubugins.^21^

Studies have shown that trace metals can be introduced into tea products during the fermentation and drying processes of production.^23^,^24^ The metallic surfaces of machinery used in grinding and squeezing water out of the leaves have also been reported to contribute to trace metal levels, resulting in the adsorption of Cu and Pb to the tea leaves.^25^ The major source of trace metals contamination is, however, considered to be the soil on which the tea plants are grown. Soil may be contaminated due to precipitation of acid rain in environments where discharge of gases of carbon dioxide and sulfur dioxide are prevalent since trace metals become more soluble, mobile and bioavailable in an acidic medium.^26^,^27^ Anthropogenic activities such as the use of wastewater for irrigation, sewage sludge, use of pesticides, agricultural applications of chemical fertilizers, mining and smelting activities, and automobile emissions are also important sources of trace metal pollution in soils.^28–31^ The determination and quantification of only the total concentrations of trace metals may be misleading since this assumes that all forms of a chemical element have equal impact on the environment or bio-systems.^32^ The biological availability and fate of trace metals for uptake in biological systems are dependent on the chemical forms in which they exist, and therefore, quantifying the total levels of trace metals is insufficient for explaining their mobility and predicting their toxicity. Hence, chemical fractionation or speciation provides a better understanding of the lability and toxic potentials of trace metals.^32^,^33^

The consumption of tea products as daily dietaries and medications has been increasing steadily all over the world. Green, black and herbal teas contain both inorganic and organic chemical substances that possess carcinogenic, mutagenic, toxic, and genotoxic properties. Human exposure to inorganic nonessential elements has been shown to result in adverse health effects. Chronic exposure to Pb levels of 1×10^−4^ μg/g/day may result in memory loss, while Cd levels above 1×10^−3^ μg/g may lead to kidney damage, weakening of the bones and fractures.^34–37^ The objectives of the present study are to evaluate the chemical partitioning and total concentrations of Cd, Cr, Cu, Mn, Ni, Pb, vanadium (V), and Zn in different green, black and herbal tea brands imported or produced in Nigeria, to evaluate any significant differences between tea brands in their trace metal contents and to compare data obtained with the metal concentrations in tea products reported in literature from other parts of the world.

**Abbreviations**
*HCl*: Hydrochloric acid
*HNO*_*3*_: Nitric acid
*MP-AES*: Microwave plasma atomic emission spectroscopy

## Methods

All reagents used in the present study were of Analar grade in quality. Concentrated nitric acid (HNO_3_) (70%) and concentrated perchloric acid (HClO_4_) (60%) were purchased from BDH Chemicals Ltd, England, concentrated hydrochloric acid (HCl) (37%) and acetic acid from Sigma-Aldrich, France and hydrogen peroxide H_2_O_2_ (30%) solutions from Scharlau, Spain, hydroxylammonium acetate (99%) from Alfa Aesar, USA, and ammonium acetate (98%) from J.T. Baker, USA. Aqua regia was freshly prepared by mixing concentrated HCl and HNO_3_ solutions at a 3:1 volume ratio. Freshly distilled de-ionized water from an EASYpure^TM^ water purification system (Barnstead Thermolyne Corp. USA) was used. The following equipment: weighing balance (AE Adam Scales, USA), pH meter – (Hanna Instruments Benchtop pH 211 Microprocessor, USA), hot air oven – (Uniscope SM9053, Surgifriend Medicals, England), Stuart heat-stir (US152) hot plate, fume cupboard, Stuart orbital shaker SSL1, and centrifuge, microwave plasma atomic emission spectroscopy (MP-AES) were used in this study.

### Sample collection and preparation

Eleven (11) green, eight (8) herbal and four (4) black tea samples commonly consumed in Nigeria were purchased from various commercial outlets in Lagos and Ogun States. The tea brands and the manufacturers' details are presented in Table 1.^19^,^20^,^38^ The size of each dried sample was reduced by the coning and quartering method and homogenized by grinding mechanically in a clean porcelain mortar and pestle.

**Table 1:** General Information on Green, Black and Herbal Tea Samples Used in the Present Study

**Tea type**	**Product name**	**Sample code**	**Country of origin**
*Black Tea*	Lipton Yellow Label Tea	LYL	Nigeria
	Top Tea (*ginger*)	TTG	Nigeria
	Top Tea (*lime and lemon*)	TTL	Nigeria
	Top Tea (*regular*)	TTR	Nigeria
*Green Tea*	Bigelow Green Tea	BGT	USA
	Gold blend Green Tea	GBG	Sri Lanka
	Heladiv Green Tea	HGT	Sri Lanka
	Lipton Green Tea (*blackberry pomegranate*)	LGB	USA
	Lipton Green Tea (l*emon and ginseng*)	LGL	USA
	Lipton Green Tea (r*ed goji raspberry*)	LGR	USA
	Lipton Green Tea (j*asmine passion with fruit*)	LGJ	USA
	Lloyd Green Sense (*aloe vera*)	LGS	Poland
	Super blend Green Tea (*vanilla*)	SBG	Sri Lanka
	Twinings Pure Green Tea	TWG	United Kingdom
	Ty-phoo Pure Green Tea	TPG	United Kingdom
*Herbal Tea*	Anti-cancer Tea	ACT	China
	Anti-hypertensive Tea	AHT	China
	Joint Care Tea	JCT	China
	Kidney Flush Tea	KFT	China
	Moringa Herbal Tea	MHT	Nigeria
	Natural Liver Flush Tea	NLF	China
	Sahul Slim Herbal Tea	SSH	India
	Tranquilizing & Brain Nourishing Tea	TBN	China

### Sequential and pseudo-total metal extraction and analysis

A modified Community Bureau of Reference method was used for the speciation of trace elements in the tea samples as previously reported.^39^ The sequential extraction procedures are as outlined below:

#### (a) Fraction 1 (F1; Soluble/exchangeable fraction/bound to carbonates)

First, 1.0 g of oven-dried sample was weighed into a 100 mL conical flask. Then 20 mL of the prepared 0.1 mol/L acetic acid was added. The mixture was shaken on a Stuart orbital shaker SSL1 at room temperature at 300 rpm for 960 minutes. The mixture was centrifuged at 4000 rpm for 20 minutes. The separated mixture was decanted by filtration into sample bottles. The residue was washed several times with distilled water, centrifuged at 3000 rpm for 10 minutes, filtered and preserved for the next step.

#### (b) Fraction 2 (F2; Reducible fraction – fraction associated with oxides of Fe and Mn)

Twenty (20) mL of hydroxyl ammonium chloride was added to the residue from F1 in a 100 mL conical flask. The mixture was shaken at 300 rpm for 960 minutes. The mixture was centrifuged at 4000 rpm for 20 minutes and decanted by filtration into sample bottles. The residue was washed with distilled water, centrifuged at 3000 rpm for 10 minutes, filtered and preserved for the next step.

#### (c) Fraction 3 (F3; Oxidizable fraction - fraction bound to organic matter)

The residue from F2 was dispersed in 5 mL of 30% hydrogen peroxide and digested at room temperature for 1 hour with occasional shaking. A second aliquot of 5 mL 30% hydrogen peroxide was added to the mixture and digested at 85°C in the water bath for 1 hour. The mixture was evaporated on the hot plate to about 2 mL. Thereafter, 25 mL of 1.0 mol/L ammonium acetate was added to the cool, moist residue. The mixture was shaken and centrifuged at 4000 rpm for 20 minutes. The mixture was decanted and filtered into sample bottles and the residue was washed several times with distilled water and preserved for the next step.

#### (d) Fraction 4 (F4; Residual fraction)

Ten (10) mL of a mixture of concentrated HNO_3_, HCl and perchloric acid in a ratio of 6:2:5, respectively, was added to the residue from F3 and agitated at 300 rpm for 5 hours at a temperature of 80°C on a Thermo Scientific MaxQ 4000 shaker. The mixture still had some particles of tea residue; therefore 2 mL of the acid cocktail was added and transferred to a hot plate at controlled temperatures between 150°C and 200°C with continuous shaking for 1 hour to obtain a clear solution.

Sequential extraction gives more detailed information about the biological, physicochemical availability, mobilization and transport of trace elements. This is because not all forms of a given metal have the same impact on the environment or living systems. Hence, the chemical form of a metal is a function of its beneficial or toxic health effects on organisms and the environment.^30^

For the pseudo-total metal extraction, samples were oven dried at 105°C for 30 minutes and mechanically milled in a clean grinding bowl with a grinding ball to homogenize and increase the surface area. Aqua regia was prepared in a 3:1 ratio of concentrated HCl and HNO_3_. Then 1.0 g of the ground tea sample was weighed into a 250 mL conical flask. Fifty (50) mL of aqua regia was added and the conical flask was transferred to a Stuart heat-stir hot plate to heat at controlled temperatures between 50°C and 250°C for about 2 hours in the fume cupboard. Twenty (20) mL of aqua regia was later added as the volume reduced until a clear solution was obtained. The cool solution was filtered and made up to 100 mL with deionized water in a 100 mL volumetric flask. The concentrations of Cd, Cr, Cu, Mn, Ni, Pb, V, and Zn were analyzed using MP-AES.

### Quality control

A suite of quality control samples including method blanks, spiked blanks, duplicate samples, and calibration standards were systematically and routinely used to monitor relative accuracy and quality of data from the MP-AES elemental determinations. Calibration standards were prepared by appropriate dilutions of commercially available stock solutions (1000 mg/L BDH grade) of the heavy metals determined. Stock solutions containing 0.5, 1.0 and 2.0 mg/L of Cd, Cr, Cu, Mn, Ni, Pb, V, and Zn were used to prepare the calibration curves. The mean recoveries of Cd, Cr, Cu, Mn, Ni, Pb, V, and Zn were 100.06±0.47%, 101.31±3.25%, 97.56±4.18%, 100.98±4.73%, 99.04±2.69%, 99.95±2.14%, 100.54±2.03%, and 100.64±8.16%, respectively. The limit of detection (LOD) and limit of quantitation (LOQ) were determined using data from the calibration curves as 3 and 10 times the standard deviation of the standards, respectively, as depicted in Equations 1 and 2 below. The calibration procedure involved three replicates for each set of measurements. The limit of detection (LOD) had values that ranged from 0.001 mg/L (Cu, Cr, Mn) to 0.01 mg/L (Ni, Pb, Zn), while the limit of quantitation (LOQ) varied from 0.002 mg/L (Cu) to 0.02 mg/L (Pb, V, Zn).

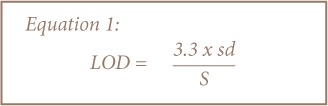


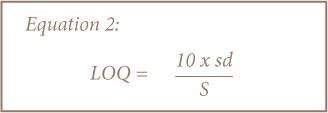
Where, sd is the standard deviation of the calibration curve, and S represents the slope of the calibration curve.

## Results

The concentrations of Cd, Cr, Cu, Mn, Ni, Pb, V, and Zn determined through sequential extraction procedure in twenty-three (23) branded tea samples (11 green tea, 8 herbal tea, 4 black tea) are presented in Tables 2 – 3. The pseudo-total metal results showed varied concentrations in green, herbal and black tea samples *(Table 4).* The concentration of Cr, Ni and Zn obtained for green and black tea samples considered in this study were comparable to levels reported in tea brands from Brazil.^4^ However, high levels of Cd and Pb were detected in green and black tea samples when compared with results reported for green and black tea products from India and Brazil.^40^,^41^ Copper, Mn and V were not detected in high levels in the tea samples investigated in the present study, and were comparatively lower than similar reported concentrations. Presently, there is no national regulation specifying the maximum allowable limits for toxic elements in tea, infused leafy beverages and herbal materials in Nigeria. However, according to World Health Organization regulation and other national (Canada, China, Malaysia, Singapore, Thailand) limits stipulated for toxic metals such as Cd (0.3 – 1.0 mg/kg), Cr (2.0 mg/kg), Cu (150 mg/kg), and Pb (10 – 20 mg/kg) in raw and finished herbal products, the levels of Cd and Cr determined in this study exceeded these established limits.^42^ Lead and Cu levels were, however, lower than the regulated limits.

**Table 2:** Summary of Concentrations (mg/kg) of Sequential Extraction Fractions (F1-F4) for Green Tea

	**HGT**	**TPG**	**LGR**	**LBG**	**LGJ**	**LGL**	**GBG**	**LGS**	**TWG**	**SBG**	**BGT**
***Cd***	4.44±0.04	3.28±0.40	3.22±0.20	4.79±0.05	2.29±0.09	2.81±0.15	4.15±0.07	4.19±0.02	2.71±0.19	3.10±0.4	3.26±0.06
***Cr***	2.30±0.05	1.40±0.03	1.91±0.03	2.55±0.03	2.55±0.04	1.12±0.03	2.03±0.03	2.19±0.01	2.31±0.01	1.87±0.02	2.17±0.20
***Cu***	1.37±0.13	1.30±0.09	1.04±0.08	1.11±0.64	0.88±0.06	0.79±0.06	0.98±0.08	0.97±0.13	1.45±0.02	1.16±0.05	1.48±0.07
***Mn***	36.75±.01	31.94±0.01	31.80±0.50	36.69±0.02	11.75±0.03	33.95±0.03	40.39±0.02	61.96±0.02	39.24±0.02	15.25±0.03	54.18±0.77
***Ni***	3.66±0.08	2.63±0.07	3.02±0.07	4.27±0.28	2.69±0.07	0.88±0.03	3.25±0.04	3.49±0.05	2.60±0.09	2.97±0.04	2.66±0.05
***Pb***	1.81±0.05	1.33±0.06	0.78±0.40	2.21±0.04	1.00±0.31	1.00±0.10	1.66±0.05	1.65±0.04	1.11±0.04	0.81±0.13	1.92±0.05
***V***	0.17±0.14	0.42±0.05	0.41±0.16	0.28±0.05	0.29±0.25	0.19±0.20	0.20±0.05	0.27±0.10	0.9±0.25	0.27±0.05	0.69±0.07
***Zn***	10.21±0.04	3.49±0.04	3.21±0.03	7.54±0.03	4.72±0.04	1.70±0.04	3.47±0.04	25.49±0.03	4.84±0.05	1.36±0.05	6.10±0.04

**Abbreviations**: BGT, Bigelow green tea; GBG, Gold blend green tea; HGT, Heladiv green tea; LBG, Lipton green tea (*blackberry pomegranate*; LGL, Lipton green tea (lemon and ginseng); LGR, Lipton green tea (red goji raspberry); LGS, Lipton green tea (jasmine passion with fruits); SBG, Super blend green tea (aloe vera); TPG, Ty-phoo pure green tea; TWG, Twinings pure green tea

**Table 3:** Summary of Concentrations (mg/kg) of Sequential Extraction Fractions (F1-F4) for Herbal and Black Tea Samples

	**Herbal Tea**								**Black Tea**			
	**NLF**	**AHT**	**TBN**	**JCT**	**MHT**	**KFT**	**SSH**	**ACT**	**LYL**	**TTG**	**TTL**	**TTR**
***Cd***	3.08±0.08	2.82±0.18	3.35±0.06	2.79±0.08	1.54±0z.10	1.98±0.34	1.95±0.26	3.17±0.08	3.03±0.07	3.05±0.04	3.07±0.05	2.81±0.07
***Cr***	1.99±0.02	2.19±0.00	1.66±0.58	2.23±0.04	2.01±0.03	2.48±0.06	2.45±0.05	2.25±0.04	1.87±0.69	2.18±0.05	2.60±0.02	2.48±0.02
***Cu***	1.62±0.04	1.65±0.04	1.76±0.11	1.61±0.03	1.44±0.04	1.96±0.06	1.39±0.06	1.45±0.07	1.35±0.06	1.44±0.08	1.35±0.03	1.38±0.04
***Mn***	32.54±0.04	41.36±0.02	59.85±0.02	52.16±0.03	2.56±0.03	25.33±0.03	3.49±0.08	59.64±0.03	48.07±0.02	48.32±0.03	37.04±0.03	40.47±0.02
***Ni***	3.07±0.08	2.64±0.15	2.68±0.08	2.60±0.02	2.17±0.23	2.52±0.14	2.51±0.08	2.83±0.02	2.86±0.03	2.84±0.06	3.23±0.08	2.02±0.05
***Pb***	1.69±0.06	0.93±0.09	0.84±0.06	1.02±0.11	0.85±0.13	0.88±0.07	0.73±0.07	1.75±0.04	1.12±0.05	1.19±0.06	1.22±0.08	1.02±0.03
***V***	0.64±0.10	0.53±0.05	0.67±0.05	0.61±0.20	0.86±0.08	0.80±0.08	0.57±0.12	0.53±0.04	0.71±0.03	0.64±0.11	0.52±0.08	0.54±0.07
***Zn***	7.60±0.03	2.24±0.03	8.22±0.02	2.03±0.03	3.18±0.03	2.01±0.04	1.41±0.04	1.86±0.04	2.25±0.04	7.42±0.03	1.12±0.07	1.33±0.03

**Abbreviations**: ACT, Anti-cancer tea; AHT, Anti-hypertensive tea; JCT, Joint care tea; KFT, Kidney flush tea; LYL, Lipton yellow label tea; MHT, Moringa herbal tea; NLF, Natural liver flush tea; SSH, Sahul slim herbal tea; TBN, Tranquilizing and brain tea; TTG, Top tea (ginger); TTL, Top tea (lime and lemon); TTR, Top tea (regular)

**Table 4:** Pseudo-total Trace Metal Concentrations (mg/kg) (mean ± Standard Deviation) in Green, Herbal and Black Tea Samples

		**Cd**	**Cr**	**Cu**	**Mn**	**Ni**	**Pb**	**V**	**Zn**
***Green Tea***	***TPG***	0.22±0.00	0.06±0.00	0.19±0.00	4.9±0.00	0.1±0.00	0.07±0.00	0.02±0.00	0.65±0.00
	***HGT***	0.43±0.03	0.09±0.00	0.31±0.01	8.26±0.33	0.16±0.1	0.13±0.00	0.03±0.00	0.74±0.04
	***GBG***	0.59±0.00	0.08±0.00	0.39±0.01	13.39±0.02	0.18±0.01	0.16±0.00	0.05±0.00	0.9±0.01
	***SBG***	0.32±0.08	0.10±0.04	0.22±0.04	4.55±0.22	0.15±0.05	0.07±0.01	0.03±0.02	0.79±0.13
	***LBG***	0.43±0.01	0.11±0.00	0.18±0.00	8.13±0.01	0.17±0.00	0.09±0.00	0.04±0.00	0.93±0.09
	***LGL***	0.57±0.19	0.13±0.04	0.17±0.01	6.77±3.91	0.4±0.37	0.11±0.03	0.03±0.01	0.63±0.11
	***LGR***	0.44±0.01	0.12±0.00	0.14±0.00	9.61±0.14	0.18±0.01	0.09±0.01	0.03±0.00	0.28±0.01
	***LGJ***	0.29±0.00	0.1±0.00	0.13±0.00	5.36±0.01	0.14±0.00	0.06±0.00	0.04±0.00	0.29±0.05
	***LGS***	0.53±0.01	0.1±0.00	0.19±0.00	14.71±0.06	0.18±0.00	0.16±0.00	0.03±0.00	0.34±0.00
	***BGT***	0.59±0.08	0.18±0.02	0.17±0.00	13.28±0.08	0.25±0.06	0.14±0.01	0.03±0.01	0.38±0.04
	***TWG***	0.51±0.04	0.14±0.01	0.17±0.00	11.28±0.07	0.21±0.02	0.11±0.01	0.03±0.00	0.37±0.08
***Herbal Tea***	***NLF***	0.64±0.00	0.18±0.00	0.18±0.00	13.67±0.02	0.42±0.00	0.12±0.00	0.01±0.00	0.46±0.01
	***TBN***	0.68±0.08	2.51±2.02	0.3±0.12	17.30±3.70	0.29±0.04	0.20±0.06	0.02±0.00	0.6±0.17
	***MHT***	0.72±0.00	0.20±0.00	0.14±0.00	0.89±0.01	0.89±0.00	0.13±0.00	0.02±0.00	0.73±0.01
	***SSH***	0.92±0.01	0.5±0.01	0.22±0.00	1.26±0.08	1.10±0.01	0.07±0.00	0.03±0.00	0.48±0.08
	***AHT***	0.55±0.03	0.13±0.00	0.25±0.00	11.87±0.36	0.30±0.00	0.11±0.00	0.01±0.00	0.55±0.00
	***JCT***	0.54±0.01	0.11±0.00	0.19±0.00	14.77±0.19	0.19±0.00	0.19±0.00	0.02±0.01	0.55±0.00
	***KFT***	0.44±0.01	0.19±0.00	0.33±0.00	7.91±0.04	0.22±0.00	0.11±0.00	0.03±0.01	0.73±0.01
	***ACT***	0.56±0.00	0.11±0.00	0.20±0.00	17.23±0.00	0.18±0.00	0.18±0.00	0.02±0.00	0.42±0.00
***Black Tea***	***LYL***	0.36±0.02	0.39±0.01	0.15±0.00	10.23±0.09	0.17±0.00	0.08±0.00	0.02±0.00	0.30±0.06
	***TTG***	0.71±0.01	0.30±0.00	0.31±0.00	8.47±0.10	0.54±0.01	0.12±0.00	0.01±0.00	0.55±0.01
	***TTL***	0.42±0.00	0.16±0.00	0.15±0.00	11.39±0.05	0.17±0.00	0.12±0.00	0.03±0.01	0.29±0.02
	***TTR***	0.53±0.00	0.27±0.01	0.19±0.00	12.43±0.09	0.19±0.00	0.17±0.00	0.13±0.00	0.42±0.03

**Abbreviations**: ACT, Anti-cancer tea; AHT, Anti-hypertensive tea; BGT, Bigelow green tea; GBG, Gold blend green tea; HGT, Heladiv green tea; JCT, Joint care tea; KFT, Kidney flush tea; LBG, Lipton green tea (*blackberry pomegranate;* LGL, Lipton green tea (lemon and ginseng); LGR, Lipton green tea (red goji raspberry); LGS, Lipton green tea (jasmine passion with fruits); LYL, Lipton yellow label tea; MHT, Moringa herbal tea; NLF, Natural liver flush tea; SSH, Sahul slim herbal tea; TBN, Tranquilizing and brain tea; TTG, Top tea (ginger); TTL, Top tea (lime and lemon); TTR, Top tea (regular); SBG, Super blend green tea (aloe vera); TPG, Ty-phoo pure green tea; TWG, Twinings pure green tea

## Discussion

### Green tea

The values of Cd, Cr, Cu, Mn, Ni, Pb, V and Zn were higher in green tea than in the investigated herbal and black teas. This is consistent with previously reported results.^22^ Cadmium had a low presence in the reducible fraction, but was evenly distributed in the soluble/exchangeable, oxidizable and residual fractions. Approximately 27% of Cr was bioavailable, i.e. present in the soluble/exchangeable fraction; a larger portion was distributed in the oxidizable and residual fraction. Copper was bound to all of the different fractions except in Gold blend green tea, where it was not present at all in the soluble or exchangeable form. Manganese was bound largely in soluble/exchangeable and reducible fractions, indicating its high mobility and bioavailability. Nickel was predominantly bound to the oxidizable and residual fractions with about 15–19% in soluble/exchangeable fractions. Lead was distributed in soluble/exchangeable, oxidizable and residual fractions with a presence of around 5–10% in the redsucible fractions. Vanadium was mainly present as oxides of Fe and Mg. Zinc was scarcely present in the bioavailable form, but highly present in the residual fraction, evidencing its high stability in the samples. High concentrations of Ni and Cr are known to have carcinogenic effects on humans. Chromium acts as a cofactor for insulin, affecting neurotransmitter levels in the human body. Lead levels may cause the accumulation of neurotoxic metabolites in the body and affect the release of dopamine, a neurotransmitter. Cadmium is extremely toxic and has the capacity to displace Zn in metallo-enzyme actions, causing Cd-induced Zn deficiency.^43^ The results also suggest that green teas are a viable source of essential trace elements such as Cu, Mn and Zn. Manganese was largely the most bioavailable and Zn was the least bioavailable in Lloyd green tea.

### Herbal tea

The herbal teas generally recorded lower trace metal concentrations compared to the green teas and Mn had the highest concentration across the samples. The herbal teas also proved be good sources of essential trace metals such as Cu and Mn. Zinc toxicity may result in nausea, vomiting, epigastric pain, abdominal cramps, central nervous system deficits and Zn-induced Cu deficiency.^44^ Cadmium in the herbal teas was predominantly bound to organic matter and as sulfides (oxidizable fraction) and smaller fractions in the soluble/exchangeable and residual fraction except for Moringa herbal tea, in which it was not detected in the bioavailable form. Chromium was distributed in the oxidizable and residual fraction with about 31% presence in soluble/exchangeable fractions. Copper was found mainly in soluble/exchangeable and residual fractions, with smaller portions in reducible and oxidizable fractions, making it about 40–45% bioavailable. Manganese was dominant in soluble/exchangeable and reducible and almost non-existent in the residual fraction. This is a pointer to its high mobility and biological availability. Nickel occurred predominantly in its oxidizable states with lower portions in soluble/exchangeable and residual fractions. Lead occurred in the order oxidizable> soluble/exchangeable≥residual>reducible fractions. Vanadium showed high stability by being present more in the residual fraction than levels that were found in the reducible and oxidizable fractions, but a sizeable portion was also found in the soluble/exchangeable form or bound to carbonates. Zinc showed high stability in Natural liver flush tea and Tranquilizing and brain nourishing tea brands, while other herbal tea samples indicated variable concentrations in all four fractions. Zinc was found to be least bioavailable in Tranquilizing and brain nourishing tea, while Mn was most mobile and bioavailable in a certain branded anti-cancer tea (ACT).

### Black tea

Among the samples, Top tea (lime and lemon flavor) recorded the highest concentrations for Cd (3.07 mg/kg), Cr (2.60 mg/kg), Ni (3.23 mg/kg) and V (0.71 mg/kg), while Top tea (ginger flavor) had the highest concentrations for Cu (1.44 mg/kg), Mn (48.32 mg/kg) and Zn (7.42 mg/kg). The highest concentration of V (0.71 mg/kg) was recorded in Lipton yellow label tea. Cadmium, Ni and Pb were present largely in the oxidizable fraction, while Cr was bound almost equally in the oxidizable and residual fractions, with a lower amount in the soluble fraction and almost non-existent in the reducible fraction. Copper was evenly distributed in soluble/exchangeable, oxidizable and residual fraction with a lower proportion in reducible fractions. Manganese was preferably bound to soluble/exchangeable and reducible fractions, indicating its high mobility and bioavailability. Vanadium and Zn were higher in the reducible fraction, an indication of their non-bioavailability. However, V was more bioavailable in herbal and black teas than in green tea. Copper stimulates the production of dopamine, which increases brain activity.^45^ It is interesting to note that the black tea samples, all of which were produced locally, recorded lower trace metal concentrations compared to the green and herbal teas. This suggests improved practices in tea plant cultivation, non-polluted growing soils and processing methods. Low concentrations of trace elements are known to be beneficial to plants. However, deficiencies in Zn may result to poor growth, sexual maturation, enlarged liver, skin rash and anaemia.^46^ Like the green and herbal tea samples, Zn was also found to be least bioavailable in Top tea (ginger flavor), while Mn was most bioavailable in Lipton yellow label tea samples.

Generally, Mn had the highest concentration in all of the analyzed samples, and was mostly found in the bioavailable fraction, indicating considerable potential of direct toxicity to the human system. This is in agreement with results from similar reported studies that showed that the accumulation of Al and Mn can be used as convenient variables and significant predicators for tea classification and discrimination.^40^,^47^ The Mn levels also suggest that tea is a significant source of Mn and people with Mn deficiencies may get the required quantities from tea consumption. The Cr levels were slightly higher than those reported in the literature.^22^ However, the Cu levels in the present study were lower than the concentrations reported by Szymczycha-Madeja et al.^22^ High levels of Zn were detected in Heladiy green tea and Top tea (ginger flavor) (black tea). Lead, Cr, Cu and V levels were found to be comparatively low in concentration. Green tea presented relatively higher values for Zn and Ni, which explains why these metals leach more in green tea than in oolong or black tea.^47^,^48^

### Pseudo-total concentrations of trace metals

The total concentration of trace metals in the branded Camellia sinensis samples showed the green teas having up to approximately 80% Mn content, followed by 7–10% Zn, 4–5% Cd, Cu and the other elements distributed in the remaining fraction. This is in agreement with previous findings.^49^ Manganese was also dominant in the herbal tea with up to 85% of the total elements, followed by Zn, Cd and Ni. The black teas showed more evenly distributed portions of trace metals with the dominance of V and Ni in Top tea (regular) and Top tea (ginger flavor), respectively. Manganese, Zn and Cd speciation can be attributed to their affinity to carbonates. This suggests that black tea is a good source of dietary trace elements. The differences in metal content distribution may be attributed to age of leaves, type of soil, location of production or country of origin. However, chronic cadmium ingestion in animals can cause an increase in systolic blood pressure even when renal disease is absent. Increased aldosterone in the blood, renal dysfunction, retention of sodium and water in the body are also harmful effects of Cd exposure.^35^

Generally, the observed sequence of trace metals accumulation is Mn>Zn>Cd>Ni>Cr>Cu>Pb>V. Similar studies did not observe any significant differences in the concentration of metals in the green and black teas.^49^,^50^ This study however, observed that Mn levels in green tea were very high, up to 80%, while for black tea samples, Mn concentrations ranged between 15% and 30%. Improved cultivation practices and processing methods for black tea may be the reason for the observed differences.

## Conclusions

The present study examined the trace metal contents of each tea type (dry Camellia sinensis and herbal tea), imported and locally produced for the Nigerian market. The sequential extraction procedure and pseudo-total analysis methods were used in determining the metal concentrations in the dry tea samples. The results showed considerable metal loads in each tea type. The data showed that manganese was the most bioavailable of the trace metals studied, being largely found in the soluble/exchangeable fraction, while Zn was most preferably bound to the residues. Other elements, including Cd, Cr, Cu, Ni, Pb, and V were distributed at various concentrations in the various fractions. Based on the results of this study, tea has proven to be a rich source of Mn and consumption of tea may be recommended to humans with Mn deficiency. Cadmium and lead levels detected in tea samples in this study followed the sequence herbal>black>green. Locally produced black tea samples recorded low trace metal contents compared to the green and herbal tea samples.
